# Connection Failure: Differences in White Matter Microstructure Are Associated with *5-HTTLPR* but Not with Risk Seeking for Losses

**DOI:** 10.3390/ijms25126666

**Published:** 2024-06-18

**Authors:** Philipp T. Neukam, Dirk K. Müller, Yacila I. Deza-Lougovski, Shakoor Pooseh, Stephanie H. Witt, Marcella Rietschel, Michael N. Smolka

**Affiliations:** 1 Department of Psychiatry and Psychotherapy, Technische Universität Dresden, 01307 Dresden, Germany; philipp.neukam@mssm.edu; 2 Institute for Medical Informatics and Biometry, Carl Gustav Carus Faculty of Medicine, Technische Universität Dresden, 01307 Dresden, Germany; 3 Institute of Psychology, University of the Bundeswehr München, 85577 Neubiberg, Germany; yacila.deza-lougovski@unibw.de; 4 Center for Interdisciplinary Digital Sciences (CIDS), Technische Universität Dresden, 01069 Dresden, Germany; shakoor.pooseh@tu-dresden.de; 5 Department of Genetic Epidemiology in Psychiatry, Central Institute of Mental Health, University of Heidelberg, 68159 Mannheim, Germany

**Keywords:** *5-HTTLPR*, risky choice, DTI, decision-making, structural connectivity

## Abstract

S/S carriers of *5-HTTLPR* have been found to be more risk seeking for losses compared to L/L carriers. This finding may be the result of reduced top-down control from the frontal cortex due to altered signal pathways involving the amygdala and ventral striatum. The serotonergic system is known to be involved in neurodevelopment and neuroplasticity. Therefore, the aim of this study was to investigate whether structural differences in white matter can explain the differences in risk-seeking behaviour. Lower structural connectivity in S/S compared to L/L carriers and a negative relationship between risk seeking for losses and connectivity were assumed. Diffusion-weighted imaging was used to compute diffusion parameters for the frontostriatal and uncinate tract in 175 genotyped individuals. The results showed no significant relationship between diffusion parameters and risk seeking for losses. Furthermore, we did not find significant differences in diffusion parameters of the S/S vs. L/L group. There were only group differences in the frontostriatal tract showing stronger structural connectivity in the S/L group, which is also reflected in the whole brain approach. Therefore, the data do not support the hypothesis that the association between *5-HTTLPR* and risk seeking for losses is related to differences in white matter pathways implicated in decision-making.

## 1. Introduction

Understanding the neurobiological basis of decision-making under risk is an important topic in the neuroeconomic community. Kahnemann and Tversky [1] showed with their prospect theory that individuals do not behave rationally when making choices involving risks but instead show a bias termed the *reflection effect*. It describes the observation that when offered a smaller but certain amount of money and a larger but probabilistic amount to gain, individuals are risk averse, i.e., they prefer the safe option. The opposite pattern, hence, *reflection effect*, is shown when the offers are about losing money, either a certain smaller amount or a larger but probabilistic amount. Here, individuals usually behave more risk seeking, i.e., they choose the probabilistic offer more often than the safe option.

This reflection effect has been suspected to stem from emotional responses that bias choices to be more risk averse or risk seeking. Indeed, using a risky choice paradigm where offers were framed either as gains or losses, De Martino et al. [2] found heightened amygdala activation when choices were made in agreement with the reflection effect (the sure option in the gain domain, the risky option in the loss domain) compared to the opposite behaviour. Further evidence from a mixed gambles task showed that the ventral striatum (VS) codes for the expected value of probabilistic choices in the gain domain and the amygdala for expected value in the loss domain [3]. In general, the striatum and medial parts of the frontal cortex are known to carry valuation signals by integrating outcome-related information, such as magnitude, probability, and delay (for reviews, see [4,5]), and some evidence suggests that the valuation process may be influenced by the amygdala [6]. Further evidence for a role of the amygdala in loss-related decision-making is provided by De Martino et al., who showed that the amygdala mediates loss-aversion behaviour possibly by computing an arousal signal related to the prospective monetary loss [7]. Moreover, the amygdala has strong connections to frontal brain regions, and recent studies show that its activity is regulated via inhibitory top-down control by the ventromedial prefrontal cortex (vmPFC), and a loss of control can result in potentiated amygdala activity [8].

In order to perform these complex decision-making steps, optimal information transportation between these brain regions via white matter tracts is crucial. In humans, white matter integrity in fibre bundles can be indirectly assessed using diffusion tensor imaging (DTI). Briefly, this technique measures the diffusion of water molecules that move freely (isotropy) in water but diffuse along white matter rather than across it, which introduces directionality in the water diffusion (anisotropy). There are four scalar parameters that are commonly calculated and extracted based on the diffusion behaviour per voxel: fractional anisotropy (FA), mean diffusivity (MD), axial diffusivity (AD), and radial diffusivity (RD). AD represents the eigenvalue of the principal diffusion direction, RD is the two eigenvalues of the diffusion directions perpendicular to the primary diffusion, MD is the average of the three eigenvalues representing mean diffusivity, and FA quantifies diffusion anisotropy, taking into account both AD and RD (i.e., the ratio of standard deviation and root mean square of the eigenvalues) [9,10,11]. In the absence of additional information, higher FA values are commonly interpreted as representing more intact white matter, while lower FA values have been associated with neuropathology, such as axonal damage due to stroke and neurodegenerative disorders [11,12]. However, FA alone does not provide information about whether a higher or lower score is related to changes in AD or RD. Therefore, these parameters are also assessed to provide additional information to support an interpretation of FA.

The *5-HTTLPR*, a naturally occurring genetic variation in the promoter region of the gene (SLC6A4) coding for the serotonin transporter (5-HTT), may have a strong influence on decision-making and white matter properties. *5-HTTLPR* primarily regulates the transcriptional efficiency (and, hence, transporter availability) in a way that one allelic variant with 14 repeats (short or S-allele) results in lower transcriptional efficiency and the other variant with 16 repeats (long or L-allele) results in higher transcriptional efficiency and, eventually, 5-HTT availability. PET imaging with the radioligand [^11^C]DASB revealed a high density of 5-HTT in the striatum, moderate-to-high in the amygdala, and moderate in the vmPFC [13,14]. It influences neuronal signalling between the amygdala, striatum, and vmPFC/OFC [15], and its availability as well as regulatory activity is also very important during the development of the central nervous system since 5-HT influences neuronal plasticity, proliferation, and differentiation [16]. There are two interesting studies that investigated the investment behaviour of S/S carriers in investment tasks and also included additional information about their real-life wealth, income, debt, and personality traits [17,18]. Overall, they found that S/S individuals were more risk averse, rather chose not to invest and had a more pessimistic belief about their wealth and financial standing, even though they did not differ from S/L and L/L carriers when objective measures, such as income and debt were considered. In line with previous studies [19,20], they showed that S/S carriers had higher neuroticism scores and reported more negative affect, which may cause an overall negative view on potential future outcome. A study that specifically included 149 investors adds to this picture by providing some evidence that S/S-allele carriers assume a lower life expectancy for themselves compared to L/L carriers, which again deviates from objective measures of average life expectancy [21]. These findings indicate that S/S carriers may have a strong bias towards avoiding potential losses and, hence, avoid risks for gains. This is corroborated by a study conducted in our lab demonstrating that when confronted with a smaller but certain loss or a larger but probabilistic loss, S/S carriers showed a stronger tendency to accept a higher probabilistic loss than a lower certain loss [22].

There is evidence that these genetic-related biases may be explained by differences in white matter tracts (see also Table 1). For example, a study used DTI to investigate white matter microstructural properties of the uncinate tract, a fibre bundle that connects the temporal lobe (including the amygdala) with the inferior frontal lobe (i.e., the vmPFC), together with a fear-conditioning paradigm to measure amygdala reactivity across 5-HTTLPR groups in 100 participants [23]. They found increased amygdala activation in S-allele carriers compared to the L/L group as well as increased FA in the S-allele participants, which they interpreted as elevated bottom-up control. There are, however, two other studies that found the opposite result, i.e., reduced FA for S-allele carriers [24,25]. It should be noted that the former study only tested 33 and the latter 37 females, which makes it difficult to draw a strong conclusion based on their findings.

Another important bundle that has been of high interest in the realm of decision-making research is the frontostriatal (also termed accumbofrontal) tract, which connects the VS with the vmPFC [26]. Especially in the delay discounting domain, several studies reported a negative relationship between FA values of this tract and temporal discounting rates in young adults [27] and developing populations in the age range of 8–25 years [28,29]. These studies suggest a relationship between the structural properties of the frontostriatal tract and delay discounting. However, to the authors knowledge, no study so far has investigated how 5-HTTLPR-related differences in the structural properties of the frontostriatal as well as the uncinate tract, two fibre bundles implicated in decision-making, relate to probabilistic choice for losses.

**Table 1:** Summary of previous studies investigating the effect of either *5-HTTLPR* on white matter tracts/functional coupling or the effects of white matter tract microstructure on behaviour.

Study	Sample	Polymorphism	Tract	Task	Results
Klucken et al., 2015 [23]	100 adults (46 females)	Triallelic *5-HTTLPR*	Uncinate Fasciculus	Fear conditioning	S-allele carriers showed increased amygdala responses during fear learning and increased amygdala–insula coupling compared to l/l-carriers. S-allele carriers had higher FA values compared to l/l-carriers.
Pacheco et al., 2009 [24]	37 females	Triallelic *5-HTTLPR*	Uncinate Fasciculus	n/a	Regression showed reduced FA with an increasing number of S-alleles
Jonassen et al., 2012 [25]	33 females	Triallelic *5-HTTLPR*	Uncinate Fasciculus	n/a	ANCOVA revealed lower FA in the left Uncinate for S/S carriers vs L/L carriers as well as a significant linear trend
Peper et al., 2013 [27]	40 adults (20 females)	n/a	Frontostriatal	Delay Discounting	Stronger discounting was associated with higher MD, RD and lower FA
Achterberg et al., 2016 [28]	192 adults (51.2% females	n/a	Frontostriatal	Delay Discounting	Age-related increases in FA were associated with reduced discounting
Olson et al., 2009 [29]	79 adults (53.2% females)	n/a	ATR, CCsp, IFOF, ILF, SLF, UF, CST	Delay Discounting	Lower discounting was associated with higher FA and lower MD

FA: fractional anisotropy, MD: mean diffusivity, RD: radial diffusivity, ATR: anterior thalamic radiation, CCsp: splenium of the corpus callosum, IFOF: inferior fronto-occipital, fasciculus, ILF: inferior longitudinal fasciculus, SLF: superior longitudinal fasciculus, UF: uncinate fasciculus, CST: corticospinal tract.

Therefore, the aim of this study was to investigate in a larger sample (1) whether there is a linear increase in FA values from S/S-allele to L/L-allele carriers in white matter bundles and (2) whether differences in individual white matter can explain higher risk seeking for losses in S/S-allele carriers. Based on the literature, the uncinate and frontostriatal fasciculus were chosen as a priori tracts of interest, as they are most likely to be involved in influencing decision-making and be modulated by *5-HTTLPR*. Consequently, a reduced structural connectivity of the uncinate and frontostriatal tract in S/S compared to L/L genotype individuals and a negative relationship between the structural connectivity (indicated by higher FA, AD and lower MD, RD) and risk seeking for losses scores were hypothesised. Finally, an exploratory voxel-based approach of the whole brain white matter was used to investigate possible relationships between other fibre bundles, genotypes, and risk seeking for losses.

## 2. Results

### 2.1. Sample Information

Demographic information is shown in Table 2. A total of 221 participants completed the study. Of those, 38 were excluded due to imaging-related artifacts (see Section 4.4), and 8 participants had missing data for the probability discounting for losses (PDL) task, resulting in 175 data sets for the analysis. Sex was not equally distributed across genotype groups (*p* = 0.027) while age varied similarly across genotype groups (*p* = 0.051).

### 2.2. Linear Contrast Results for 5-HTTLPR and White Matter Tracts

**Figure 1:**
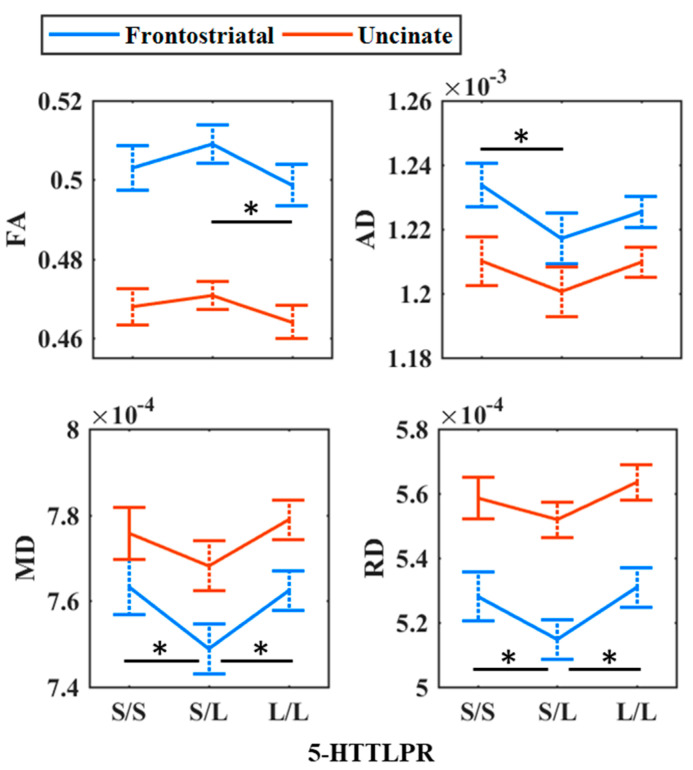
Main effect of *5-HTTLPR* on DTI parameters: fractional anisotropy (FA), axial diffusivity (AD), mean diffusivity (MD), and radial diffusivity (RD). Error bars are bootstrapped with 10,000 iterations and denote 95% bias corrected and accelerated confidence intervals. * *p* < 0.05.

Simple linear contrast analyses between S/S and L/L groups did not reveal any significant differences for each of the DTI metrics for both the frontostriatal (all *p* > 0.06) and uncinate (all *p* > 0.28) tract. See Table 3 and Figure 1 for details. The results also did not change when we combined the S/S and S/L group.

### 2.3. Exploratory Nonlinear Contrast Results for 5-HTTLPR and White Matter Tracts

For the frontostriatal tract, the nonlinear multivariate analysis of covariance (MANCOVA) showed a significant main effect of the genotype (Wilk’s Λ = 0.924, F6,330.000 = 2.222, *p* = 0.041, ηp² = 0.039). A subsequent one-way ANCOVA with Games–Howell post hoc tests showed that S/L individuals had significantly higher FA values than L/L individuals (*p* = 0.013), S/S had higher AD values compared to S/L individuals (*p* = 0.007), while S/S and L/L carriers had higher MD (pS/S = 0.004; pL/L = 0.002) and RD values (pS/S = 0.024; pL/L = 0.001) compared to S/L carriers. These results indicate higher structural connectivity in S/L carriers compared to the two homozygous groups (Figure 1).

For the uncinate fasciculus, the MANCOVA revealed significant main effects of sex (Wilk’s Λ = 0.953, F3,165.000 = 2.689, *p* = 0.048, ηp² = 0.047) and age (Wilk’s Λ = 0.952, F6,330.000 = 2.222, *p* = 0.041, ηp² = 0.039). Post hoc independent sample t-tests demonstrated that the effect of sex was related to higher FA (t173 = −2.529, *p* = 0.012) and lower MD (t173 = 2.962, *p* = 0.003) and RD (t173 = 2.888, *p* = 0.004) but not AD (t173 = 1.593, *p* = 0.113) in males compared to females. Finally, Pearson’s correlations showed that age was significantly negatively correlated with AD (r = −0.219, *p* = 0.004) but not with FA (r = 0.005, *p* = 0.948) or MD (r = −0.143, *p* = 0.059) nor RD (r = −0.073, *p* = 0.336). Descriptive statistics are shown in Table 4.

### 2.4. Correlations between the DTI Parameters of the Frontostriatal/Uncinate Tracts and Risk Seeking for Losses

**Figure 2:**
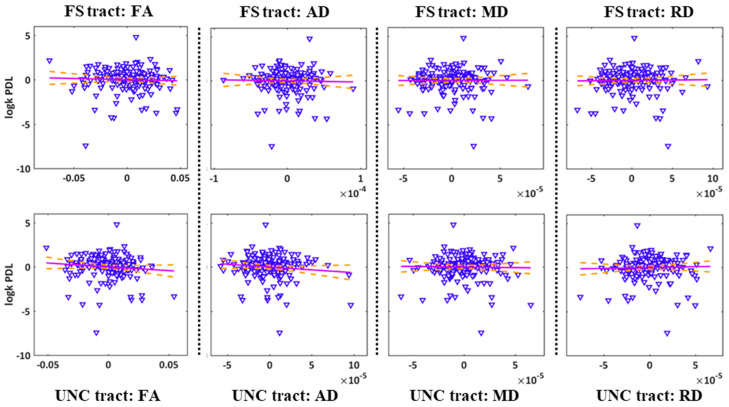
Scatter plot of correlations between risk seeking for losses (log*k* PDL) and DTI parameters: fractional anisotropy (FA), axial diffusivity (AD), mean diffusivity (MD), and radial diffusivity (RD). There were no significant relationships between log*k* and DTI parameters. All DTI parameters are unstandardised residuals after controlling for 5-HTTLPR, sex, and age. The purple lines show linear trends and the dashed lines indicate 95% confidence intervals.

The results are depicted in Figure 2. Correlations between risk seeking for losses (log*k* PDL) and DTI parameters: fractional anisotropy (FA), axial diffusivity (AD), mean diffusivity (MD), and radial diffusivity (RD). All DTI parameters are unstandardised residuals after controlling for 5-HTTLPR, sex, and age. Dashed lines indicate 95% confidence intervals. Pearson correlations revealed no significant correlation between the PDL discounting parameter log*k* and the four diffusion metrics, FA, MD, AD, and RD (all *p* > 0.14).

### 2.5. Exploratory Whole Brain Results

**Figure A1:**
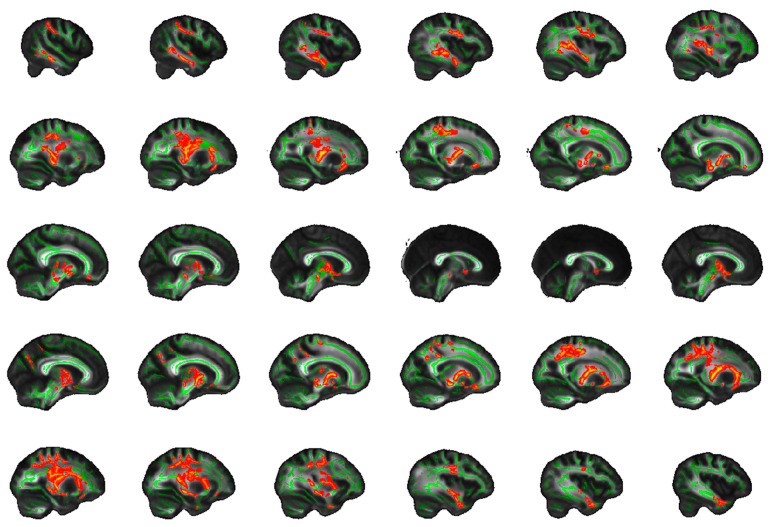
The TBSS main effect of *5-HTTLPR* based on 10,000 permutations for fractional anisotropy, where red denotes a weaker and yellow a stronger effect of *5-HTTLPR.* All voxels survive a family-wise error correction of *p* < 0.05.

**Figure A2:**
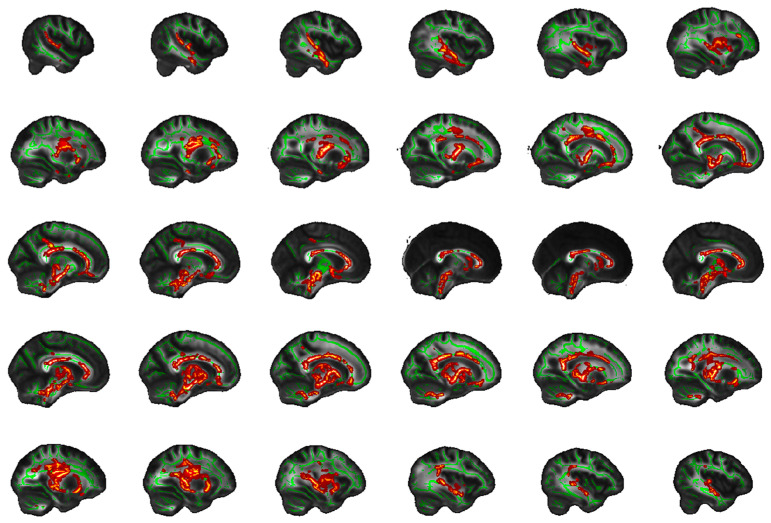
The TBSS main effect of *5-HTTLPR* based on 10,000 permutations for axial diffusivity where red denotes a weaker and yellow a stronger effect of *5-HTTLPR*. All voxels survive a family-wise error correction of *p* < 0.05.

**Figure A3:**
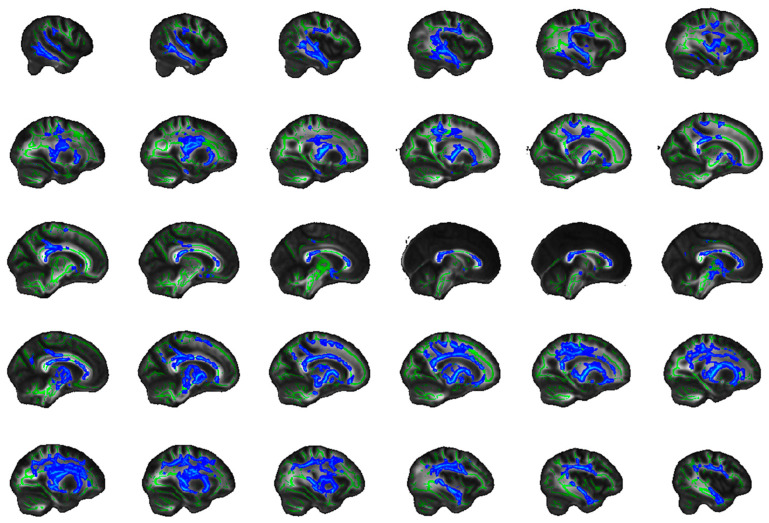
The TBSS main effect of *5-HTTLPR* based on 10,000 permutations for mean diffusivity where dark blue denotes a weaker and light blue a stronger effect of *5-HTTLPR.* All voxels survive a family-wise error correction of *p* < 0.05.

**Figure A4:**
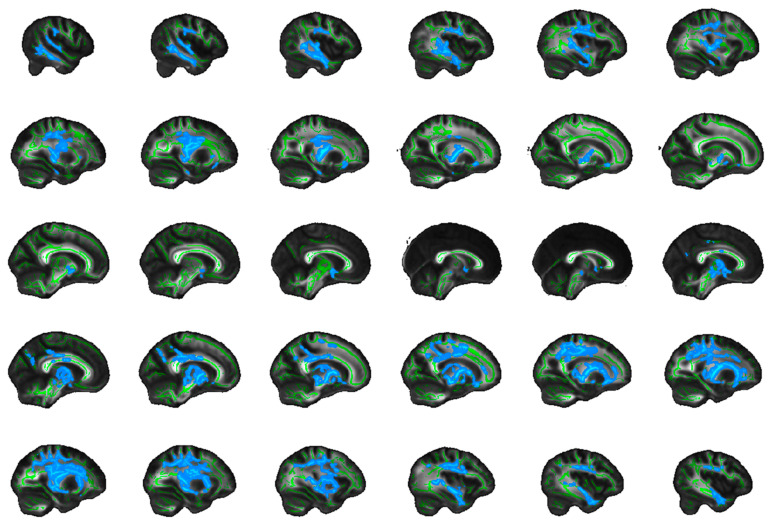
The TBSS main effect of *5-HTTLPR* based on 10,000 permutations for radial diffusivity where dark blue denotes a weaker and light blue a stronger effect of *5-HTTLPR.* All voxels survive a family-wise error correction of *p* < 0.05.

The results for the main effect of the genotype are summarised in Table 5. There were no significant clusters for the main effect of PDL, nor for the interaction with genotype. Visual depictions of the main effect of the genotype are shown in Appendix A, Figure A1, Figure A2, Figure A3 and Figure A4.

Post hoc analyses in the context of each of the four MANCOVAs showed that S/S individuals had higher FA values (M_S/S_ = 0.48 ± 0.018, M_L/L_ = 0.47 ± 0.017, *p* = 0.020, Cohen’s d = −0.434) and lower RD values (M_S/S_ = 5.44×10^−4^ ± 2.21 × 10^−5^, M_L/L_ = 5.47 × 10^−4^ ± 2.46 × 10^−5^, *p* = 0.021, Cohen’s d = 0.414) compared to L/L individuals.

## 3. Discussion

The purpose of this study was to elucidate whether the association between risk seeking for losses, as measured with a PDL task, and *5-HTTLPR* may be explained by differences in white matter connecting brain regions that are involved in value-based decision-making (vmPFC, VS, amygdala). Based on the existing literature, we chose the frontostriatal and uncinate tract. The former connects the VS with the vmPFC and the latter the vmPFC with the amygdala. Interestingly, the frontostriatal tract has been implicated in decision-making behaviour but not in *5-HTTLPR*, while the opposite is true for the uncinate tract. This study extends on these findings by examining the association of *5-HTTLPR* with both tracts and furthermore by investigating the relationship between the tracts and risk seeking for losses.

The results of all data analyses can be summarised in two parts. First, the DTI parameters (FA, AD, MD, RD) are not related to the discounting rates of the PDL task, neither in the tracts of interest nor with whole brain white matter. Therefore, differences in white matter structure cannot explain risk seeking for losses in our sample. Second, we did not find the expected linear relationship between genotype and DTI parameters (i.e., higher FA, AD and lower MD, RD for L/L compared to S/S carriers) in the frontostriatal, in the uncinate tract, or in other white matter bundles. Hence, we could not replicate previous findings indicating reduced structural connectivity in S/S compared to L/L carriers [24,25].

### 3.1. White Matter and Risk Seeking for Losses

There are few reports that studied the contribution of white matter microstructure to value-based decision-making. The majority of existing studies focused on intertemporal choice and found negative correlations between white matter microstructure (such as the frontostriatal tract) and delay discounting (i.e., higher structural connectivity and reduced temporal discounting rates) in longitudinal studies examining participants ranging between 8 and 26 years [28,29] and young adult populations ranging between 18 and 25 years [27,30], but see [31] for an opposite finding. Much less is known about the relationship between risk seeking for losses and the uncinate fasciculus. The main motivation to select this tract was that it denotes an important pathway connecting the amygdala to the vmPFC. Research in humans and mice indicated that the frontal cortex regulates the amygdala by reducing its activation in the wake of negative events [8,32]. Therefore, reduced structural connectivity may be associated with reduced top-down control, higher amygdala activity, and, finally, increased risk seeking for losses [2].

However, our findings do not support the conclusion of the studies investigating the frontostriatal tract that higher impulsivity (steeper discounting) is associated with reduced structural connectivity in the context of risk seeking for losses. It is tempting to speculate that age may be a reason for our null finding, as all previous studies had much younger samples. Karlsgodt et al. [33], for example, showed that the frontostriatal tract microstructure (i.e., FA) increases steadily during childhood until the early twenties and stabilises and slowly decreases around the age of forty. Given that our participants were in their early 30s on average, we have not been able to capture developmental aspects of the decision-making related white matter, in contrast to the studies above, which may have contributed to our null finding. Still, as there are currently no directly relatable data published, it seems premature to draw a final conclusion on whether our finding is a true or false negative.

### 3.2. 5-HTTLPR and White Matter

Previous studies have shown interest in understanding the white matter microstructure of the uncinate fasciculus in relation to the *5-HTTLPR* because numerous studies using functional and morphometric measures suggest that S-allele carriers have increased amygdala activity [34,35], reduced grey matter volume [14,36], and a reduced coupling of the amygdala to the frontal cortex [36] compared to L/L carriers. This is in line with the hypothesis that there is a gene–dose effect, where the gene function increases with the number of L-alleles [37,38]. Such a relationship was found in two studies investigating the uncinate fasciculus that showed increasing FA values with the number of L-alleles [24,25]. Due to the observation that S/S and S/L individuals have similar 5-HTT expression rates and also score similarly on behavioural measures such as trauma exposure [39], neuroticism [19], and depressive symptoms [40], studies combine S-allele groups (S/S, S/L) and compare them to L/L carriers. This has been studied by Klucken, et al. (2015) [23], who found the opposite pattern of FA values (S > L/L) but did not find any association for the genotype and FA in a replication study [41]. This latter finding is in line with our observation that the genotype does not significantly affect FA or AD, MD, and RD in the uncinate tract, and the fact that Jonassen et al. [25] and Pacheco et al. [24] only analysed 33 and 37 females, respectively, limits the generalizability of their studies. Additionally, Klucken et al. [23] found the opposite in 100 participants containing both sexes in a previous study and no genotype effect in their replication study including 114 participants, and finally, our null finding with 175 participants supports the notion that the genotype effect is either very small or depends on other presently unknown third variables [41].

We also did not find the expected genotype effect, i.e., reduced structural connectivity in S/S compared to L/L carriers, in the frontostriatal tract. Instead, we found nonlinear effects of the genotype in AD, MD, and RD demonstrating less MD in S/L compared to S/S and L/L carriers, less AD in S/L compared to S/S carriers, and lower RD in S/L compared to L/L carriers. These findings are not intuitive and are at odds with a gene–dose effect. An explanation may be a larger proportion of individuals showing molecular heterosis in our sample, a phenomenon that describes heterozygosity in a given genetic polymorphism can result either in a greater expression (positive heterosis) or lesser expression (negative heterosis) of a phenotype compared to homozygosity, and such an observation may occur in up to 50% of all human genetic association studies [42]. In the case of *5-HTTLPR*, there is research reporting such findings in the context of 5-HTT binding potential or 5-HTT availability, where S/L individuals had lower scores compared to S/S and L/L [43,44]. Furthermore, Malmberg et al. [45] reported that male S/L adolescents had higher scores for disruptive behavioural disorders, and Steffens et al. [46] observed higher white matter volume lesions in geriatric depressed patients in comparison to the homozygous groups. Our results are in line with these observations, but the mechanisms behind heterosis are not yet understood. Comings and MacMurray [42] suggest three possible reasons: the first being an (inverted) U-shape function indicating that both too little or too much expression has adverse consequences and only intermediate expression is advantageous; the second being an independent third factor causing a hidden stratification of the sample such that in one set S/S carriers have the highest/lowest phenotypic expression and in the second set L/L carriers have the highest/lowest phenotypic expression. The third reason may be greater fitness in heterozygous individuals because they show a broader range of gene expression compared to the homozygous groups. Nevertheless, given that this is the first study reporting such a finding with DTI parameters in the frontostriatal tract, more studies are needed to support this finding.

### 3.3. Limitations

One possible limitation is that our diffusion-weighted imaging sequence was not sensitive enough to find correlations between the DTI parameters and risk seeking for losses as well as the expected linear relationship with *5-HTTLPR*. However, despite the fact that we only collected data from 32 direction, whereas newer sequences acquire data from twice our number or even more directions, we believe that our number of directions is sufficient to estimate the tensor model and, importantly, to replicate an often published finding that males show consistently higher FA and lower MD and RD compared to women in the frontostriatal and uncinate tract (while the results of AD are inconclusive), which is in line with previous findings that males have a higher structural connectivity compared to females in several brain regions [47,48,49]. Nevertheless, future studies would benefit from estimating more complex diffusion models, such as NODDI [50], which informs us about the microstructural complexity of axons and dendrites but could not be estimated in these data because it requires multi-shell imaging. Another limitation is that we apply the triallelic *5-HTTLPR* model, which subdivides the L-allele into a high expressing L_A_ and a low expressing L_G_ variant. The L_G_ allele has been found to have a similar transcriptional efficiency to the S-allele [37] and may have provided more insight regarding the reliability of the heterosis effect.

## 4. Materials and Methods

### 4.1. Participants

This study was part of two larger projects that investigated the role of dopamine and serotonin on meta-control parameters and brain function [22,51,52,53,54,55]. The recruitment was conducted via standardised invitation letters sent to addresses based on a random sample stratified by sex and age (20–40 years), which were provided by the residential registry. Individuals who passed the screening for neurological or psychiatric disorders were screened and excluded if one of the following criteria applied: pregnancy; not fulfilling the common criteria for MR safety; a current somatic disease requiring medical treatment; any psychiatric disorders that required pharmacological treatment within the last year; and a lifetime history of one of the following conditions (for ICD-10): organic psychiatric disorders (F0), opiate, cocaine, stimulants, hallucinogens, inhalants or poly-substance dependence, schizophrenia or related personality disorders (F2), and affective disorders (F3). Participants who passed the screening were invited to the study; their visual acuity was checked to ensure that it was at least 0.8. In total, 611 participants completed a baseline visit, in which blood was taken to be genotyped for the *5-HTTLPR* and stored at −81 °C until further processing. Risk seeking for losses was measured with a probability discounting for losses task using the value-based decision-making (VBDM) battery [56]. Afterwards, participants were re-invited to take part either in the dopamine or serotonin project, during which diffusion-weighted images were acquired. The local ethics review board of the Technische Universität Dresden approved of the study protocols, and all participants gave written informed consent in line with the Declaration of Helsinki.

### 4.2. Probability Discounting for Losses (PDL) Task

In this task, participants had to choose between two offers: a smaller certain loss or a larger probabilistic loss, both simultaneously presented. All offers presented were randomly shown on the left or right side on the computer screen, and the chosen offer was indicated with a red frame. Participants were informed beforehand that one of their choices in every task would be selected randomly and deducted from the total balance they could accumulate during the baseline visit. During the task, they did not receive feedback about the outcomes of each choice. Based on individual choices, the discounting rate (*k*) was estimated assuming hyperbolic discounting, following this formula [56]:

V = L/(1 + *k* ϴ) (1)

where ϴ = (1 − P)/P is the transformation of reward probability P (2/3, 1/2, 1/3, 1/4, and 1/5) to odds against winning. The loss, L, ranged from –5 Euro to −20 Euro. The task consisted of 30 trials. In all tasks, the likelihood of choosing between the two offers followed a softmax probability function in which β > 0 served as a consistency parameter such that its large values corresponded to a high probability of taking the most valuable action. In general terms, the algorithm seeks to determine the individual indifference point, i.e., finding offers where participants would theoretically decide equally between the certain or probabilistic loss. To this end, the algorithm starts from liberal prior distributions on the parameters and, after observing a choice at each trial, updates the belief about the parameters using the Bayes’ rule P(k, β|choice) ∞ P(choice|k, β)P(k, β) to find offers close at the individual indifference point. The estimated *k* parameter from the final trial best explains choice behaviour. High *k* values indicate increased risk seeking as higher, but probabilistic losses are discounted more and hence preferred over smaller, certain losses. A detailed description of the mathematical framework is reported in Pooseh et al. [56]. All tasks were implemented in MATLAB (Release 2010a, The MathWorks, Inc., Natick, MA, USA) and the Psychtoolbox 3.0.10 based on the Psychophysics Toolbox extensions [57,58].

### 4.3. Genotyping

The collected blood samples were sent to the Central Institute of Mental Health in Mannheim, Germany, to perform the genotyping for the *5-HTTLPR*. The exact procedure is described elsewhere [59]. Due to the failure to take blood from 9 participants, blood samples from 602 participants were available for genotyping. The observed allele frequency was 39.9% for S and 60.1% for L, with the following genotype groups: 99 S/S, 283 S/L, and 220 L/L. The allele and genotype frequencies did not deviate significantly from the Hardy–Weinberg equilibrium (χ^2^ = 0. 2461, df = 1, *p* = 0.62).

### 4.4. Imaging

The MR setup was the same for both studies. Diffusion-weighted images (DWI) were acquired on a 3 Tesla Magnetom TrioTim scanner (Siemens Healthcare GmbH, Erlangen, Germany), equipped with a 32-channel head coil. In total, 36 transverse scans, consisting of 4 non diffusion-weighted (B0) and 32 non-collinear diffusion-weighted images with a b1000 s/mm² factor, were obtained. Parallel imaging was realised with a GRAPPA factor = 2, while the other parameters were as follows: repetition time (TR) = 9200 ms; echo time (TE) = 92 ms; a basis resolution of 128 × 128 × 72 mm^3^ with 2.1 mm isotropic voxels (no gap); field-of-view (FOV) = 275 × 275 mm². Additionally, a high-resolution T1-weighted magnetization prepared rapid acquisition gradient echo (MP-RAGE) image for normalization, anatomical localization, and screening for structural abnormalities by a neuroradiologist (TR: 1900 ms; TE: 2.26 ms; flip angle: 9°; FOV: 256 × 256 mm²; 176 sagittal slices; voxel size: 1 × 1 × 1 mm³) was acquired. Preprocessing of the DWI data included motion and eddy current correction using FSL (version 5.0.11) eddy [60] as well as the –*repol* setting to detect and replace slices that could be considered outliers using default settings [61], all embedded in the in-house developed NICePype software (version 1.0) [62]. All 221 volumes were visually inspected for artefacts. In total, 38 data sets had to be excluded: 26 because of strong absolute rotation ≥1° along the x-, y-, or z-axis; 2 showing abnormally large ventricles; and 10 showing severe distortion artefacts. The pre-processed images were then loaded into the ExploreDTI toolbox (version 4.8.6) [63], and the diffusion tensor model was fitted to the data using the implemented RESTORE algorithm (version 4.8.6) [64]. Afterwards, the DT metrics of interest were computed: FA, MD, AD, and RD. Next, deterministic whole brain tractography [9] was performed (minimum FA = 0.2, minimal fibre length = 30 mm, maximal fibre length = 300 mm, maximum angle = 30°, cubic interpolation).

### 4.5. Frontostriatal and Uncinate Fibre Tract Selection and Volume of Interest (VOI) Generation

#### 4.5.1. Uncinate Fasciculus

**Figure 3:**
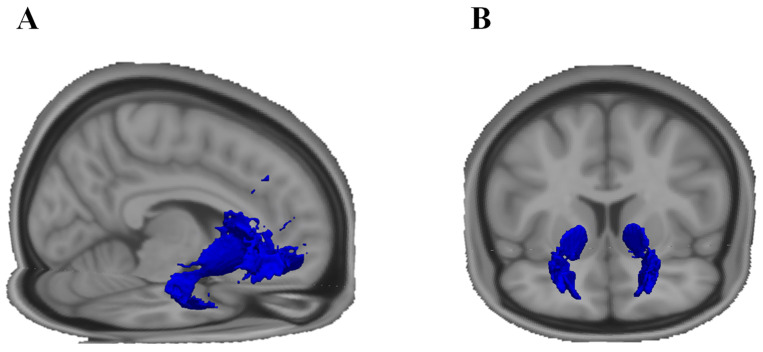
The volumes of interest (blue) of the (**A**) uncinate fasciculus and (**B**) frontostriatal fasciculus. Both volumes were created in the standard space where the uncinate tract was created based on an atlas template, and the frontostriatal tract was created based on individual tractography. Only voxels in deep white matter were analysed based on the FA skeleton created with TBSS. See Section 4.5.2. for details.

To obtain a VOI for the uncinate fasciculus, we employed a similar method to the one reported by Schaeffer et al. [65]. To this end, we used the TBSS pipeline to warp all individual DTI images to MNI space (FMRIB58 template). The warped FA images were thresholded to include only voxels with a value of at least 0.2 or above. We took the uncinate fasciculus from the John Hopkins University white matter tractography probability atlas [66] and thresholded the probabilities to greater or equal to 5% to exclude less likely voxels. Finally, each normalised and thresholded FA image was combined with thresholded probabilistic tract map, and the mean FA skeleton was computed during the TBSS procedures described above. The resulting VOIs were then retransformed to native space and applied to the DTI images to extract the parameters of interest. The VOI is shown in Figure 3A.

#### 4.5.2. Frontostriatal Tract

As the frontostriatal tract is not yet part of white matter atlases, we used a region of interest (ROI) approach, combined with the individually computed tractograms. First, we generated an ROI of the striatum by combining the accumbens, putamen, and caudate parts from the Harvard–Oxford subcortical atlas implemented in FSL. Next, we used regions (Frontal_Sup_Orb, Frontal_Med_Orb, Rectus) from the automated anatomic labelling atlas [67] to create a vmPFC/OFC mask. In order to transform the ROIs from MNI standard space to individual diffusion space, a series of computations were performed. The first step was to register the individual T1 image to MNI space using the *flirt* and *fnirt* algorithms, thereby obtaining the nonlinear transformation coefficients of interest. The next aim was to average and skull-strip the B0 images and register the averaged image to the individual T1 image using *flirt*. The obtained transformation matrix was then used with *applywarp*, together with the nonlinear transformation coefficients to warp the B0 image to MNI space. As we were interested in having the inverse matrices to transform the ROIs from MNI to diffusion space, we used to *convert_xfm* and *invwarp* operations to invert the transformations from MNI to T1 space and from T1 to diffusion space. The inverted matrices and the *applywarp* command were then used to transform the striatum and vmPFC/OFC masks from MNI to diffusion space.

The individual tractograms and transformed ROIs were next loaded into TrackVis version 0.6.1 [68] and streamlines that pass from the striatum to the vmPFC/OFC or vice versa were generated. Exclusion masks were individually set on the mid-sagittal plane, on the coronal plane at the splenium of the corpus callosum, and on the axial plane on the level of the anterior temporal gyrus. If necessary, single spurious streamlines were additionally manually removed. The resulting tracts were then exported as nifti files.

A prerequisite for the creation of a group template was the employment of the FSL tract-based spatial statistics (TBSS) pipeline [69], which warps individual FA maps to MNI space (the FMRIB58 template provided by FSL) and averages all FA images to compute a mean FA image, which is then reduced to a skeleton, based on voxels from the nearest tract centre. In a next step, all tracts were nonlinearly warped to the MNI template, registered to the FA skeleton using the *tbss_non_fa* command from TBSS and binarized. Finally, all tracts were summed up into one nifti, normalised by the number of participants, thresholded to contain only voxels that exist in at least 50% of the sample, and binarized again. This group VOI was then retransformed to native space and applied to the DTI metrics of interest (FA, MD, AD, RD) to extract the tract-related metrics. The VOI is depicted in Figure 3B.

### 4.6. 5-HTTLPR and White Matter Tracts Linear Contrast Analysis

To test our first hypothesis that the S/S genotype is associated with reduced structural connectivity compared to the L/L genotype in a linear fashion, we computed simple linear contrasts for FA, MD, AD, and RD separately for each tract while controlling for sex and age in SPSS 25 (IBM-SPSS, Chicago, IL, USA). For the second hypothesis that higher risk seeking for losses is linearly associated with reduced structural connectivity, we used partial Pearson correlations to investigate the relationship between the *k* values (on log scale to approximate a normal distribution) from PDL and the DTI parameters, controlling for *5-HTTLPR*, sex, and age. Significance was assumed at a *p*-value of <0.05.

### 4.7. 5-HTTLPR and White Matter Tracts: Exploratory Nonlinear White Matter Tracts Analysis

To explore nonlinear relationships between *5-HTTLPR* groups, white matter structure in the frontostriatal and uncinate fasciculus VOIs, and risk seeking for losses, we used a multivariate analysis of covariance (MANCOVA) for each tract with FA, AD, MD, and RD as dependent variables; with genotype (S/S, S/L, L/L) as group factor and logarithm of *k* from the PDL task as covariate of interest; and with sex and age as control variables in SPSS 25 (IBM-SPSS, Chicago, IL, USA). We set our statistical threshold of significance at *p* < 0.05.

### 4.8. Exploratory Whole Brain Analysis

To explore effects of *5-HTTLPR* and risk seeking for losses in other regions of the brain, we used the TBSS pipeline described above to skeletonise all FA, MD, AD, and RD images. We used voxelwise non-parametric statistical analyses based on 10,000 random permutations and the threshold-free cluster enhancement (TFCE) approach to test for the main effect of the genotype (S/S, S/L, L/L), the main effect of risk seeking for losses, and interaction effects. Additionally, age and sex were demeaned and entered as covariate regressors, as they were found in previous studies to be related to the DTI parameters. We assumed significance at a family-wise error-corrected *p*-value of <0.05. Classification of tracts the clusters belong to was performed with the JHU White Matter Tractography Atlas [66]. To further explore the contribution of each genotype to all significant clusters, binarized masks were generated from them, and the four DTI parameters were extracted and averaged across cluster voxels separately for each parameter. In a next step, a multivariate ANOVA was conducted with the four DTI parameters as dependent variables and genotype as predictor. 

## 5. Conclusions

Overall, we did not find a significant correlation between the white matter parameter and risk seeking for losses in two highly relevant fibre bundles or the expected association with respect to *5-HTTLPR.* However, we found evidence for the potential existence of heterosis in the frontostriatal tract that needs validation from future studies.

## Figures and Tables

**Table 2:** Demographic information.

	N (Females)	Age (SD)
S/S	39 (14)	32.3 (5.9)
S/L	56 (12)	34.8 (4.3)
L/L	80 (35)	32.7 (5.6)
Statistic	χ^2^ = 7.252, *p* = 0.027	F_2,169_ = 3.027, *p* = 0.051

**Table 3:** Results of the simple linear contrast analyses.

	S/S		L/L		Contrast Estimate	*p*-Value
	Mean	SD	Mean	SD		
Frontostriatal						
FA	0.50	0.02	0.50	0.02	−3.80 × 10^−3^	0.369
AD	1.23 × 10^−3^	2.22 × 10^−5^	1.23 × 10^−3^	2.22 × 10^−5^	−9.40 × 10^−6^	0.060
MD	7.63 × 10^−4^	2.00 × 10^−5^	7.62 × 10^−4^	2.13 × 10^−5^	−2.16 × 10^−6^	0.611
RD	5.28 × 10^−4^	2.40 × 10^−5^	5.31 × 10^−4^	2.85 × 10^−5^	1.46 × 10^−6^	0.780
Uncinate						
FA	0.47	0.01	0.46	0.02	−0.004	0.278
AD	1.21 × 10^−3^	2.44 × 10^−5^	1.21 × 10^−3^	2.15 × 10^−5^	−1.76 × 10^−6^	0.715
MD	7.76 × 10^−4^	1.95 × 10^−5^	7.79 × 10^−4^	2.08 × 10^−5^	1.944 × 10^−6^	0.639
RD	5.59 × 10^−4^	2.08 × 10^−5^	5.63 × 10^−4^	2.50 × 10^−5^	3.797 × 10^−6^	0.407

FA = Fractional Anisotropy, AD = Axial Diffusivity, MD = Mean Diffusivity, RD = Radial Diffusivity, SD = Standard Deviation.

**Table 4:** Descriptive statistics for the *5-HTTLPR* genotype groups and the frontostriatal/uncinate tract.

	S/S			S/L			L/L		
	Mean	(SD)	N	Mean	(SD)	N	Mean	(SD)	N
Frontostriatal									
Males									
FA	0.503	(0.020)	25	0.511	(0.016)	44	0.503	0.021	45
AD	1.23 × 10^−3^	(2.53 × 10^−5^)	25	1.22 × 10^−3^	(3.18 × 10^−5^)	44	1.22 × 10^−3^	(2.40 × 10^−5^)	45
MD	7.60 × 10^−4^	(2.30 × 10^−5^)	25	7.48 × 10^−4^	(2.00 × 10^−5^)	44	7.58 × 10^−4^	(2.03 × 10^−5^)	45
RD	5.26 × 10^−4^	(2.70 × 10^−5^)	25	5.13 × 10^−4^	(1.92 × 10^−5^)	44	5.26 × 10^−4^	(2.58 × 10^−5^)	45
Females									
FA	0.503	(0.016)	14	0.502	(0.026)	12	0.494	(0.026)	35
AD	1.24 × 10^−3^	(1.20 × 10^−5^)	14	1.22 × 10^−3^	(2.37 × 10^−5^)	12	1.23 × 10^−3^	(1.96 × 10^−5^)	35
MD	7.68 × 10^−4^	(1.22 × 10^−5^)	14	7.53 × 10^−4^	(2.95 × 10^−5^)	12	7.68 × 10^−4^	(2.16 × 10^−5^)	35
RD	5.32 × 10^−4^	(1.78 × 10^−5^)	14	5.22 × 10^−4^	(3.53 × 10^−5^)	12	5.37 × 10^−4^	(3.08 × 10^−5^)	35
Total									
FA	0.503	(0.018)	39	0.509	(0.018)	56	0.499	(0.024)	80
AD	1.23 × 10^−3^	(2.22 × 10^−5^)	39	1.21 × 10^−3^	(3.01 × 10^−5^)	56	1.23 × 10^−3^	(2.22 × 10^−5^)	80
MD	7.63 × 10^−4^	(2.00 × 10^−5^)	39	7.49 × 10^−4^	(2.22 × 10^−5^)	56	7.62 × 10^−4^	(2.13 × 10^−5^)	80
RD	5.28 × 10^−4^	(2.40 × 10^−5^)	39	5.15 × 10^−4^	(2.35 × 10^−5^)	56	5.31 × 10^−4^	(2.85 × 10^−5^)	80
Uncinate									
Males									
FA	0.470	(0.015)	25	0.471	(0.010)	44	0.466	(0.018)	45
AD	1.20 × 10^−3^	(2.14 × 10^−5^)	25	1.20 × 10^−3^	(3.07 × 10^−5^)	44	1.21 × 10^−3^	(2.28 × 10^−5^)	45
MD	7.68 × 10^−4^	(1.85 × 10^−5^)	25	7.67 × 10^−4^	(2.02 × 10^−5^)	44	7.75 × 10^−4^	(1.95 × 10^−5^)	45
RD	5.52 × 10^−4^	(2.09 × 10^−5^)	25	5.51 × 10^−4^	(1.72 × 10^−5^)	44	5.60 × 10^−4^	(2.28 × 10^−5^)	45
Females									
FA	0.465	(0.014)	14	0.468	(0.023)	12	0.461	(0.021)	35
AD	1.23 × 10^−3^	(1.97 × 10^−5^)	14	1.20 × 10^−3^	(2.27 × 10^−5^)	12	1.21 × 10^−3^	(1.97 × 10^−5^)	35
MD	7.90 × 10^−4^	(1.21 × 10^−5^)	14	7.71 × 10^−4^	(2.90 × 10^−5^)	12	7.83 × 10^−4^	(2.19 × 10^−5^)	35
RD	5.71 × 10^−4^	(1.42 × 10^−5^)	14	5.55 × 10^−4^	(3.35 × 10^−5^)	12	5.68 × 10^−4^	(2.71 × 10^−5^)	35
Total									
FA	0.468	(0.015)	39	0.471	(0.013)	56	0.464	(0.019)	80
AD	1.21 × 10^−3^	(2.44 × 10^−5^)	39	1.20 × 10^−3^	(2.90 × 10^−5^)	56	1.21 × 10^−3^	(2.15 × 10^−5^)	80
MD	7.76 × 10^−4^	(1.95 × 10^−5^)	39	7.68 × 10^−4^	(2.21 × 10^−5^)	56	7.79 × 10^−4^	(2.08 × 10^−5^)	80
RD	5.59 × 10^−4^	(2.08 × 10^−5^)	39	5.52 × 10^−4^	(2.14 × 10^−5^)	56	5.63 × 10^−4^	(2.50 × 10^−5^)	80

FA = Fractional Anisotropy, AD = Axial Diffusivity, MD = Mean Diffusivity, RD = Radial Diffusivity, SD = Standard Deviation.

**Table 5:** The summary cluster map of the TBSS results for the main effect of *the 5-HTTLPR*.

Tracts	Side	Peak Voxel (MNI)			F-Statistic (TFCE)	Cluster Size > 100 (Voxels)	Cluster *p*-Value
		x	y	z			
FA							
SFOF	Left	−22	−2	19	19.5	10,610	0.001
ILF	Right	45	−11	−27	14.2	6452	0.003
UNC, IFOF	Right	18	24	−12	13.8	669	0.015
AD							
Unclassified	Left	−10	−1	−14	17.3	12,958	0.001
ILF	Right	40	−22	−21	11.5	1739	0.014
Forceps minor	Right	12	31	8	10	1522	0.028
UNC, IFOF	Right	28	14	−10	9.84	786	0.03
Unclassified	Right	1	10	14	7.64	188	0.047
Forceps minor	Left	−12	29	−12	9.1	157	0.042
SLF	Left	−34	−37	21	10.3	141	0.038
ATR, IFOF	Right	23	26	23	5.94	141	0.047
MD							
ATR	Left	−11	−17	−2	18.1	31,113	0.001
RD							
ATR	Left	−23	−2	17	18.8	14,709	0.001
ILF	Right	45	−10	−28	15.3	6641	0.004
UNC, IFOF	Right	18	24	−12	13.4	209	0.039

TFCE: Threshold-Free Cluster Enhancement; FA = Fractional Anisotropy; AD = Axial Diffusivity; MD = Mean Diffusivity; RD = Radial Diffusivity; SFOF = Superior Fronto-Occipital Fasciculus; ILF = Inferior Longitudinal Fasciculus; UNC = Uncinate Fasciculus; SLF = Superior Longitudinal Fasciculus; ATR = Anterior Thalamic Radiation.

## Data Availability

Data set available on request from the authors.
